# Hospital and Institutional News

**Published:** 1912-10-05

**Authors:** 


### HOSPITAL AND INSTITUTIONAL NEWS.

### A MEDICAL OFFICER ON THE DRAFT POOR-LAW ORDER.

WE have received a very interesting letter from a workhouse medical officer of standing, in which he criticises the draft Poor-Law Order, the terms of which, in so far as they laid down the duties of medical officers, we considered in a leading article last week. Naturally enough the writer points out how objectionable it is that the superintendent nurse should be subject to the directions of the workhouse master, a point which lay beyond the immediate purview of the article, and we entirely endorse his argument for the complete exclusion of both workhouse master and matron from the administration of the sick wards. With the wisdom of experience, moreover, "Workhouse Medical Officer" has been scrutinising the duties of the workhouse master with even more care than those assigned in the draft order to his own office. The result of this scrutiny is the list of criticisms contained in his letter, to which our readers will turn with great interest. The truth appears to be that there can be no satisfactory solution of the difficulties, both administrative and medical or nursing, until the separation of the sick from every workhouse is made complete, and until that be secured there is no hope but in a reasonable interpretation of existing or draft rules and regulations. Alas! this is what all experience teaches the rules themselves preclude, and that is why we are glad to give publicity to the amendments suggested in "Workhouse Medical Officer's" letter, and shall welcome a discussion of them on the part of our readers.

### SWANSEA HOSPITAL AND WAITING CASES.

IT is not often the chairman of the house committee feels it his duty to make complaints, but such was the case at a meeting last week of the board of the Swansea General Hospital. Colonel Morgan, chairman of the house committee, in speaking to a minute calling the attention of the honorary medical staff to allegations of delay in attending casualty cases, said that the real facts were known to everybody. The casualty ward, he proceeded, was supposed to be opened at 9 o'clock every morning, but at that time no doctors attended. He knew that this was not their fault because their presence was required in the operating theatre, but the fact remained that they did not attend the casualty ward till 12 o'clock. He suggested that one member of the staff should be deputed to attend during the interval. Colonel Morgan's speech was followed by one from Mr. W. W. Holmes, who referred in general terms to the case of a man who had been brought to the hospital on the previous evening. He had not had time, he added, to confirm the complaint of two hours' delay which had been alleged. It was decided to investigate this case. The chairman of the board, Dr. Le Cronier Lancaster, said that it was impossible to deal satisfactorily with general statements, but the staff were prepared to investigate specific cases. Judging from the statements made at the meeting, and taking them at their face value, it would seem that here is an example of the unfortunate reports which get circulated because arrangements have been allowed to get out of date. While sympathising with the staff view as put forward by Dr. Le Cronier Lancaster, we would point out that it is advisable for the staff to insist on specific cases being brought forward so that the charge of delay may be dealt with once and for all. Everything indicates that a modification of existing hours, on the lines of Colonel Morgan's suggestion, is all that is needed.

### THE CASE AGAINST THE SUNDAY CINEMATOGRAPH.

WE have always argued against the expediency of hospitals attempting to benefit from Sunday cinematograph shows on the general ground that those institutions which sought to do so would have to face the very heavy responsibility of any disaster to the Sunday Fund movement which might occur and before long be very obviously laid on them. If that statesmanlike view of the situation was too far-seeing for a few hospitals where self-interest tempted them to take shorter views, it may be worth while to supplement it from the figures as to profits which were published last week. An analysis of these figures appears to show that the trade received in nine months £55,000, while the profits to charities were £15,000 only. This is enough to prove once more how the charities who were approached in the matter were merely used as a stalking-horse by the trade. There is also the nature of the contracts entered into to be considered. As Mr. F. W. Drewett, the director of the Prince of Wales Hospital at Tottenham, pointed out some time ago, the risks attaching to the hospitals as lessees for the Sunday performances were very heavy indeed, and in the event of the risk becoming an actuality would give to the charity concerned an unenviable notoriety into the bargain. The case, then, against hospitals attempting to benefit from Sunday cinematograph shows is a comprehensive one, and we take this opportunity of summing up the arguments lest there are any to whom the general principle at stake is not sufficiently understood, as it certainly should be by all intelligent men.

### A NEW DEVELOPMENT OF THE ALMONER'S WORK.

THE latest almoner's appointment is that of Miss Sketchley, who has been trained under the auspices of the Hospital Almoners' Council, and has been appointed lady almoner at the General Lying-in Hospital, York Road, S.E. She had the advantage of training in the maternity department of St. Thomas's Hospital, which has a high reputation for the careful supervision of this class of patient. This is the first appointment in this country of an almoner to a lying-in hospital, though there is, perhaps, no kind of institution in which an almoner can more usefully be employed. Other lying-in hospitals, no doubt, will follow the example which has been set them in this matter.

### A HOSPITAL EXPERT OF MUNICH.

The death was announced last month of one of the best-known and most popular hospital experts on the Continent. This was Dr. Pachmayer, a Magistratsrat of the City of Munich, and chief councillor of the directorate of the city hospitals. Dr. Pachmayer was a prominent official in the city life of Munich, and was appointed Verwaltungsrat of the city hospitals in 1900. Under his fine management and direction arose the extensions which have made the Munich municipal hospitals second to none in Bavaria, and rivalled in completeness and thoroughness alone by those of Berlin and Hamburg. Pachmayer was a charming host and cordial friend, and hospital workers who were privileged to make his acquaintance will remember his cheery optimism which triumphed over every suggested or real difficulty. It was this cheerfulness that made him so popular at the 1905 Congress of German hospital workers held at Munich, and that increased his wide circle of friends at the Karlsruhe Conference in 1910. He took an extremely lively interest in hospital affairs, and was one of the executive of the German Hospital Association. One of his ambitions was to make the 1913 Congress of the Association, which is to meet at Munich, of international nature by inviting representatives from other Associations to attend. The Congress will miss much through his absence, and the hospitals of Munich will have cause to regret his decease. He died, as he had often expressed a wish to die, in harness, succumbing to an attack of apoplexy while he was busied with his administrative work in his private office at Rathaus.

### STRAWBERRIES 7s. 6d. A POUND.

It would have been surprising if the lay press had not found a catchword or an incident in connection with the Birmingham Conference to serve as headline to some much-condensed report of more than average interest. Mr. Danvers Power, in his able paper on "Hospital Responsibility," provided the necessary stimulus by telling the story of the physician who ordered a patient strawberries at 7s. 6d. per pound, and the secretary who refused to sanction the order. We are now waiting for the usual discussion as to whether the secretary was right or wrong in so refusing and whether or not strawberries are medicine! Our readers need not be reminded of the fact that no profession is free from faddists, the scientific professions least of all, and that there are doctors who do not mind prescribing anything which is empirically considered useful in relieving symptoms. The duty of the hospital secretary is to use his discretion in allowing or disallowing such prescriptons, and no sensible person will quarrel with him in prohibiting strawberries at a time when the fruit was scarcely obtainable, and when potted or bottled fruit, medicinally as efficacious in this special connection, could have been procured at far less expense. Very often an expensive drug is ordered, but in that case the secretary will hesitate before he refuses his sanction. In all such cases, however, we believe that whatever difficulty may arise can easily be settled by referring the matter to the medical committee. Where the secretary is himself a medical man he naturally has a greater check upon the individual propensities of his staff, which is, perhaps, an added argument in favour of the view that every hospital should have a medical superintendent.

### A WINDFALL FOR THE SWANSEA HOSPITAL.

The Swansea General and Eye Hospital benefits to the extent of £10,000 under the will of the late Mr. Grahame Vivian, of Clyne Castle, near Swansea. The institution is one of the oldest in the West, dating from 1814, when it was established as a dispensary. In 1817 it was converted into an infirmary, and in 1867 into a general hospital, with which, nearly thirty years later, the old Swansea Eye Hospital was incorporated. The hospital has a twenty-bed convalescent home at Cwmdonkin Park and a good Samaritan fund.

### A POINT IN INTERNATIONAL LAW.

An interesting point has been raised by Dr. Fremantle, the Medical Officer of Health for Hertfordshire. As a major in the medical department of the Territorials, he is a divisional sanitary officer in the East Anglican Division of the Force, and is, therefore, in intimate contact with the sanitary companies which form part of the Divisions. At a meeting of the United Services Medical Society Dr. Fremantle pointed out that apparently the wording of the Geneva Convention of 1906 will not protect the members of sanitary companies or the Royal Army Medical Corps men employed on water duties in the event of hostilities taking place. The words of the clause protect those "engaged exclusively in the collection, transport, and treatment of the wounded and sick, as well as in the administration of medical units and detachments." Since the object of the sanitary companies and the R.A.M.C. *personnel* "for water duties" is the prevention of disease, not the cure of it, the Convention as it stands would seem not to apply to them. Yet it certainly ought to do so, for the prevention of avoidable suffering on the battlefield is surely as important as its relief. It would be well if this point were borne in mind and brought forward on the next occasion of the revision or renewal of the Geneva Convention by the signatory Powers.

### A MODEL ANTI-PHTHISIS SCHEME.

The State Sickness Insurance Committee of the British Medical Association has drawn up a model scheme for the treatment of tuberculosis, with the general practitioner as its pivot. The various districts will have a chief medical officer, whose time will be spent in administrative duties, and the object, as aimed at by a series of forms, is to insist on every tuberculous patient having a private doctor to take continuous interest in his case. In consultation with him the chief officer will decide whether domiciliary, sanatorium, dispensary, or hospital treatment shall be adopted. In the latter event on discharge the patient should bring a report to his private doctor. The "continuous record" insisted on by the Local Government Board Order of July 26

will be kept, and a fee of not less than 5s. will be due to the private practitioner for it. No patient is to be seen at the dispensary without the recommendation of a private doctor, and the work done at the dispensaries should be purely diagnostic, consultative, bacteriological, and statistical in character.

### PRESERVATIVES IN CREAM.

The regulations forbidding the use of preservatives in milk as well as in cream which comes below a specified standard are none the less welcome because of their belated appearance. Eleven years have elapsed since a Departmental Committee recommended regulations of a character similar to those which came into force on Tuesday this week, and during that time preservatives have been employed to an unnecessary and excessive extent. Cream containing 35 per cent. and upwards of milk fat may still be preserved with boric acid, borax, or hydrogen peroxide provided the consumer is informed that the cream is preserved, but the inclination to add an excess of boric acid will be checked by the necessity of declaring the percentage used, so that producers are encouraged to vie with each other in reducing the amount to the lowest possible limit. During the winter months none at all may be needed, and it will be interesting to see how little boric acid will suffice in the hot weather. In all probability these regulations will be followed by others dealing with various classes of foodstuffs, for the employment of chemical agents which will prevent the development of the micro-organisms concerned in putrefaction is too extensively practised. They render it possible to preserve articles of food in incipient decomposition with every appearance of freshness, and the sooner the Local Government Board turns its attention to other over-preserved foodstuffs the better for the public health.

### MEDICAL APPLIANCES UP TO DATE.

The London Medical Exhibition has been in progress from Monday to Friday this week at the Royal Horticultural Hall. The stands, which numbered more than one hundred, were of more than usually varied description, and the exhibits included an operation theatre in miniature, *x*-ray apparatus, apart from the inevitable and innumerable preparations of every kind. Certain features of the Exhibition have been introduced no doubt to appeal to the lay public and its excitement-loving ears, for which purpose the obesity apparatus and the "icycle" are likely to prove very successful. The gastroscope is still "a draw," and it would be impossible to mention every appliance which human ingenuity has here gathered together. The hospital officer and the practitioner, however, who have attended have probably escaped these features, going with a previous definite knowledge of the type of instrument they wished to view.

### A PRISON DOCTOR ON CRIME.

Dr. Goring, one of the medical officers at Parkhurst Prison, has drawn up a statistical report of the criminals of English prisons, in which he adduces observations which discredit the theories, associated with Lombroso, that an index of criminality may be found in the shape of an ear, or the peculiarity of a toe. This criticism of a theory that, like others advanced by the same school of psychiatrists, tried to prove too much, and was prepared to state even what particular crime might be predicted of a Roman nose, or whether homicide was a corollary of hammer toes, will surprise few intelligent observers. These will ask in what Dr. Goring's positive affirmations consist. Broadly, he denies the association of physical character with mental bias to all but patently apparent cases, and states that the criminal is not differentiated in any marked manner by physical or mental anomalies from the ordinary respectable citizen. This should be greeted with delight by those who have insisted, in spite of all the charges of sentimentalism so constantly hurled against them, that environment rather than inherent disposition is the main causative factor in leading to a life of crime. They will not feel any less shaken in this view, probably, when Dr. Goring goes on to diagnose a constitutional incapacity due to weakness of will in the criminal classes, for these qualities are obviously not confined to criminals, and in all but extreme instances a man may escape social disaster if his environment is favourable enough to protect him from the worst consequences of these propensities. The public schools as well as the police courts can show many boys whose natural want of power is only not criminal in the sense that circumstances are so favourable as to save its appearance, the demands made upon its possessor being extremely small.

### MEDICINE IN POLITICS.

The next Parliamentary election for Southampton, which is now represented by two Liberals, will see a medical candidate, Dr. E. H. Stancomb, who will stand in the Labour interest, and has gained the support of the National Administrative Council of the Independent Labour Party. We welcome this fact on the general ground of the desirability of strengthening medical representation in the House of Commons, in which the lawyers have had all their own way for a number of years. It will be noted with some interest that Dr. Stancomb is the second medical candidate who has appeared in the past quarter—his predecessor being Mr. J. H. H. Williams, L.S.A., who was unsuccessful in the by-election at East Carmarthenshire a month ago. Both men, it will be remembered, have been put forward in the Labour interest, and it would seem as if medicine in politics became identified with the party representing the poorer classes in the State. At all events, it has been this party which of late has had the greater share in the small but increasing number of medical men now entering the field of politics. Dr. Stancomb, a resident in his proposed constituency, is an Edinburgh man, and graduated at the University M.B., C.M. in 1881. He is a town councillor in the locality.

### JOINT ACTION BY POOR-LAW GUARDIANS.

Lord Richard Cavendish, in presiding at the North-Western Poor-Law Conference at Southport, struck an important note in referring to the steps that have been taken at Chester to utilise two workhouses for the reception of two distinct classes of inmates—namely, feeble-minded children and adults respectively. Now, Lord Richard rightly pointed out that the importance of this was not to be measured by their ability to form a joint scheme for the whole area, but by the foundation which the scheme laid for future joint enterprises by Boards of Guardians. The day is quickly coming when one standard of a progressive Board will be whether or not it is associated in some joint enterprise with others. Even if that be regarded as a paradoxical expression of the truth, the statement put from the opposite point of view becomes almost a truism. The most valuable schemes which Poor-Law Guardians have achieved have been schemes which they have jointly undertaken. Only two weeks ago we had occasion to speak of the example of Monyhull Colony for Epileptics, the phenomenal success of which has been possible only by the joint action of three Boards in the Birmingham area. We hope that Lord Richard Cavendish's words will be acted on by all who attended the North-Western Conference, for we have already witnesses to their importance at Monyhull and elsewhere.

### THE LATE HEAD OF THE NAVAL HOSPITALS.

The Inspector-General of Hospitals and Fleets, Royal Navy, Sir Herbert Mackay Ellis, died on Monday last at the age of sixty-one. He had seen a fair amount of active service, having entered the Navy as a surgeon in 1875. Seven years later he served with the Royal Marines in Egypt, was present at the Battle of Tel-el-Kebir, and was promoted to Staff surgeon. In 1891 he became Fleet surgeon, and was on board the unfortunate *Victoria* in his official capacity when that vessel was sunk after a collision with the *Camperdown* off Tripoli in 1893. In 1899 Sir Herbert, who, however, was not awarded the K.C.B. till 1907, became Deputy Inspector-General, rising to the senior post in 1904. In 1908 he retired from active service. He was an F.R.C.S., and an honorary physician to King Edward and King George.

### RESIGNATION DAY AND ITS RESULTS.

Resignations of club practice were general on Saturday last in London and in many of the chief towns of the provinces. In the capital every one of the sub-districts into which the area has been divided showed an almost unanimous compliance with the directions of the British Medical Association. Indeed, nothing perhaps is more symptomatic of the general agreement than the pledge not to accept any contract practice which has been resigned by fellow-practitioners, that has almost invariably accompanied the non-resignation of members. As typical of the solidarity of the profession in the provinces it may be mentioned that in Manchester and Salford not more than eight medical men will retain the appointments which they have previously held. Almost similar figures are returned from Liverpool, and the present estimate of 4 per cent. recalcitrant doctors shows that in no case is the opposition anywhere but numerically small, and thus if these accepted the terms offered by the Government it is felt that they would be confronted with such numbers of persons as would make their responsibility on the face of it overwhelming and unworkable.

### SPECIALIST AND HOSPITAL FOUNDER.

It is not only as an aural surgeon that the death of Dr. Edward Woakes demands attention, though to the general public who remember him before his retirement in 1904, this was, of course, his chief claim to reputation. In those days, indeed, he was senior aural surgeon to the London and Golden Square Hospitals, and his treatment of nasal polypus really was that of a specialist in the true sense of the word. But apart from these things he was a founder of hospitals. After qualifying in London, he joined his father at Luton, and, not content with general practice, he established the Luton Cottage Hospital, which is now known as the Luton (Bute) institution, in 1872. Since then it has been rebuilt and enlarged on separate occasions, and is now an institution containing forty beds. The honorary secretary is Mr. H. O. Williams. Four years after it was founded Dr. Woakes returned to London, and his Harley Street life began. Not content with this, however, though doubtless as an outcome of it, he assisted in founding the London Throat Hospital in Great Portland Street, which was established in 1887. Of its work, including post-graduate lectures and demonstrations, there is of course no need to speak, but his double interest in the hospital and professional side of his life is worth recording as it is not by any means a concomitant of a specialist's career.

### THE GROWTH OF OPEN-AIR SCHOOLS.

Few things have been more remarkable of late than the growth of open-air schools all over the country. We have had to chronicle in the course of the past twelve months the inception of two new ones in different parts of London, and the progress of the country hospital in the North. Now comes news of the opening of another school in Carnarvonshire by Colonel Lloyd Evans, the chairman of the County Council. The architect to the Carnarvonshire Education Authority has designed it, and on the south side is a marching corridor, the glazed screens of which can be opened in such a way as to add part of the corridor to each classroom. Bay-windows form the outer walls. It is to be noted, however, that success has been achieved without any special expenditure, as at the Paddington School, for instance, where a private house, with, it must be added, an unusually large garden for that part of London, was adopted merely in quite simple fashion for school purposes. We should not be surprised, however, if the new Carnarvon school shares the experience of others, and finds that its good work by day is in part undone by the less desirable conditions which await the children on their return home at night.

### THE POLICY OF THE PHARMACEUTICAL SOCIETY

The School of Pharmacy began its seventy-first winter session on October 2, when Mr. C. B.

Allen, the president, presented the Pereira Medal to the winner (Mr. T. H. Stroud), and Mr. J. Rymer Young, a former president of the Society, delivered the inaugural address. He said that the policy of the Pharmaceutical Society from the very first had been educational, and during the early years of its existence that policy was one which commended itself to high and low in the calling. He was sadly afraid it was not universally so now. A growing number were clamouring for what was known as a "bread-and-butter policy," which, being interpreted, meant a policy of light exertion and quick returns. It was common knowledge, he said, that not more than 25 per cent. of the chemists in business found it possible to extract a decent living out of pharmacy pure and simple, and the result was that the majority had to augment their incomes by "side-lines."

### RED CROSS FIELD-DAY AT ALNWICK.

A practical demonstration in rendering first aid to the injured removed from the battle-field has been held recently in the Duke of Northumberland's Park in connection with the British Red Cross Society. The Duchess of Northumberland and a large company watched the proceedings. A battle was supposed to have taken place, and the "wounded" were carried to two temporary hospitals erected on the field, and for final treatment were carried to the base hospital, formerly a hospital used by the 3rd Northumberland Fusiliers when encamped at Alnwick. Twenty Scouts, under Scoutmaster Goodman, acted as patients. Commandant Baker-Cresswell gave able assistance with his sectional officers. Dr. Clark Burman was examining officer, and took great pains with the students. Great thanks are due to Lady Victoria Percy from all the members of the branch. She is herself a most enthusiastic worker, and it is chiefly owing to her ladyship that the demonstration was so successful.

### THE ADMINISTRATION OF THE CHELTENHAM HOSPITAL.

The result of the local heart-searchings as to the administration of Cheltenham General Hospital, which have been alluded to in these columns, has been a special meeting which was held last week to consider a resolution which was moved by the treasurer, the Rev. R. H. M. Bouth, and included the following points: That the branch dispensary be closed and the building let or sold; that one in-patient ticket be given for each two guineas subscribed; that the present house committee, monthly and quarterly boards be superseded by a board of management having all the powers of the above, the members of which shall retire annually at the annual meeting, but shall be eligible for re-election; that the qualification for a member of the board shall be a subscription of two guineas a year. These drastic sounding recommendations were discussed at some length, but it was decided to appoint a committee "to consider how best to improve the financial and general position of the hospital." The committee will consist of the Mayor, Mr. Margrett, the Rev. R. H. M. Bouth, Mr. Geidt, Mr. Horsley, Dr. Buckell, Mr. Schuster, Colonel Cardew, Dr. Johns, and Mr. C. Williams. The paper read before the recent Conference of the British Hospitals Association, and published in our issue this week, on "Hospital Management considered with Reference to Responsibility" will perhaps give the committee some ideas as to the causes of dissatisfaction.

### A LATE FOUNDER OF PONTYPOOL HOSPITAL.

As one of those to whose enterprise Pontypool and its district owes the present hospital, the death of Dr. Samuel Mason deserves notice, apart from the suddenness with which it occurred. He was a member of its medical staff, and he leaves to his sons, Dr. George Mason and Dr. Harry Mason, the practice which his energies has built up. His previous appointments included that of resident medical assistant to the London Hospital, and he was medical officer to the local union and Poor-Law districts. He had been in the neighbourhood for forty-two years, and was actively connected with the Volunteer movement. His continued active interest in the hospital may be judged from the facts that he was one of the trustees and a member of the board, whose services in both capacities will make his loss a real one to the institution and to the neighbourhood.

### OCTOBER 12, 1912.

The above is the date of Hospital Saturday this year, and the authorities of the Fund are making an effort to retrieve the progressive rate of income, which has been showing a slight decline. They point out that the recent steady progress of the Fund has been largely the result of penny-a-week collections in the businesses and workshops of London, collections which have been more generally adopted. Since the annual street collections were done away with, a considerable sum has been raised quietly by the courtesy of tradesmen, who have kept boxes or sheets in their shops. An appeal is being made for the extension of this system.

### THE BISHOP'S WELL.

A most interesting discovery was reported to the last meeting of the managers of the Glasgow Royal Infirmary. During certain reconstruction works a well was discovered, believed to be that of the Bishop's Palace, the ruins of which were demolished in 1792 to make room for the infirmary buildings. This Palace was a thirteenth-century edifice, which at one time was fortified. The report of the master of the works, Mr. John Woodward, states that immediately under the entrance steps a well has been found about five feet square, arched over with concrete and lined on two sides with oak boards. The well has been filled in nearly up to the top, a brick wall built to catch surface water, and a pipe put in to drain the water away. An iron pump stands at the east side, and it is suggested that probably this was used to pump out surface water before the brick wall was built. If further investigation confirms the first opinions upon this ancient relic, we trust that measures will be taken for preserving so interesting an archæological monument.

### AN INDIAN HOSPITAL SCHEME.

The Maharaja of Kotah has celebrated the visit of the Queen to that State by presenting to Lady Hardinge a lakh of rupees, to be spent for the benefit of the women of India. The money is to be devoted to the provision of medical aid, and a women's medical college and hospital is to be founded at Delhi, where Indian women of the higher classes may be trained for the medical profession. It is believed that this is the only possible method of procuring sufficient practitioners for Indian women, many of whom will not be attended by men doctors. Fifteen lakhs of rupees are needed to start the scheme, and half that amount has already been subscribed by other ruling chiefs. If the requisite money is raised it is hoped to obtain the Royal permission to call the institution the "Queen Mary Medical College and Hospital."

### THE LATE SIR JOHN ELLIS.

Sir John Whittaker Ellis, who died on September 20 in his eighty-fourth year, was Father of the Court of Aldermen until 1909, when he resigned after forty-five years of active civic service. Sir John, who was created a baronet in 1882, was chairman of Emanuel Hospital and a recipient of the Order of Mercy, which was awarded him by Queen Victoria on the special recommendation of the late King Edward, then Prince of Wales. He was the first Mayor of Richmond in 1890-91, and also sat in Parliament as representative for the Mid-Surrey Division. While he never identified himself prominently with hospitals, he took an interest in their support. Among the institutions which for various reasons had won his personal support was chiefly the City of London Hospital for Diseases of the Chest, of which he was a vice-president and life governor, and at whose thirty-fourth anniversary festival he presided in 1882. He was also a life governor of the Mount Vernon Hospital and of St. Bartholomew's and St. Thomas's Hospitals (holding the latter positions ex-officio as a member of the Common Council) and a governor of the various City dispensaries.

### ALTONA'S NEW HOSPITAL.

Hamburg has already established for herself a place in the front rank of hospital cities by building and equipping three large city hospitals at a combined cost of nearly a million and a-half sterling. The sister city of Altona is now going to follow suit, but on an even more ambitious scale. The Altona Town Council has recently floated a loan of £200,000, which will be used for the building of a new city hospital. We understand that this sum will be devoted entirely to the buildings, purchase of site, and equipment of the hospital, and that no part of it will be set aside for endowment, the hospital being destined to rate support. The plans of the new hospital have not been drawn up, and it is unlikely that the preliminary specifications will be completed before next spring. The hospital, we understand, is to be a general hospital, but the style has not been settled, as much will depend on the capabilities of the site. Most probably, however, the pavilion-block compromise will be chosen as in the case of the newest Hamburg hospital. It is worth while to note that the municipality has determined to build a special children's hospital as well. This institution is being erected at Tresckowallee, in the modified pavilion style, and will be ready for occupation in the autumn of 1915. The plans present several interesting features—notably in the disposition of the annexes—which we hope to describe in detail on a future occasion.

### A RED CROSS SURGEON'S REMINISCENCES.

Mr. Henry Rundle, F.R.C.S., consulting surgeon to the Royal Portsmouth Hospital, has recently published in book form his reminiscences of the Franco-German War, in which he served as a Red Cross surgeon upon the victorious side. All the profits from the sale of this book are to be given to the building fund of the Nurses' Home at Portsmouth Hospital, and we trust that the fund may be substantially added to by this generous assistance. The book is published by Werner Laurie, and we hope to publish a review of it in a later issue. Meanwhile, we congratulate Mr. Rundle on his happy thought, and the Royal Portsmouth Hospital on a friend who, after a long career of service, is still mindful of the comfort of the nursing staff, to whose devotion so large a share of the surgical successes achieved at the hospital is due.

### THIS WEEK'S DRUG MARKET.

There has been no appreciable improvement in business during the week, and the Drug market continues quiet. The high values of opium, ergot of rye, balsam tolu, hydrastis canadensis, saffron, otto of roses, and other drugs are well maintained, and a further advance in the price of santonin is announced. The demand for cod-liver oil is not very brisk, but dealers are not disposed to give way in the matter of price. The position of glycerin is uncertain, but it is thought that any change in value would be in an upward direction. An advance in the price of antimony, which has taken place, may be followed by a rise in the quotations for antimony salts. High prices are quoted for chamomiles.

### TO OUR READERS.

Contributions are specially invited from any of our readers to these columns. They should deal with topical subjects and news. They must be authenticated for the information of the Editor only. The minimum payment if published is 5s. There is no hard-and-fast rule as to space, but notes of about twenty lines in length are preferred.

